# Indicators of Neuromuscular, Metabolic and Perceptual Fatigue Following a 5 km Run

**DOI:** 10.3390/sports14070262

**Published:** 2026-06-25

**Authors:** Klara Findrik, Petar Šušnjara, Danijela Kuna

**Affiliations:** 1 Department of Kinesiology, Faculty of Kinesiology Osijek, Josip Juraj Strossmayer University of Osijek, 31000 Osijek, Croatia; kfindrik@kifos.hr; 2 Faculty of Kinesiology, University of Zagreb, 10000 Zagreb, Croatia; 3 Department of Interdisciplinary Sciences, Faculty of Kinesiology Osijek, Josip Juraj Strossmayer University of Osijek, 31000 Osijek, Croatia; psusnjara@kifos.hr

**Keywords:** isometric strength, metabolic stress, muscle thickness, neuromuscular fatigue, recreational runners

## Abstract

High-intensity 5 km running offers an ideal framework to analyze the organism’s multidimensional responses. Since previous research primarily analyzed isolated aspects of fatigue, this study aimed to examine the integrated acute neuromuscular, metabolic, and perceptual responses to a 5 km run. Twenty-one recreational male runners participated. Pre- and post-race assessments included body composition, blood lactate, m. rectus femoris ultrasound thickness, quadriceps maximal voluntary isometric contraction (MVIC), heart rate, perceived exertion (Borg CR10), and 5 km finish time. Statistical analysis was performed in the Jamovi software, utilizing descriptive statistics, the Shapiro–Wilk test of normality, the Wilcoxon signed-rank test with effect size calculation, and Spearman’s correlation coefficient, at a significance level of *p* < 0.05. Post-race measurements revealed a significant decrease in quadriceps MVIC (pre: 305 ± 99 N vs. post: 259 ± 88 N; *p* = 0.002) and an increase in blood lactate (pre: 0.8 ± 0.4 vs. post: 6.9 ± 1.4 mmol/L; *p* < 0.001), alongside high average heart rates (165 ± 16 bpm). However, ultrasound-assessed muscle architecture remained unchanged. The 5 km run induced pronounced neuromuscular and metabolic fatigue. Unchanged muscle architecture suggests that acute strength decline is primarily mediated by metabolic and neural mechanisms, rather than immediate structural–morphological factors. These findings highlight the value of an integrated assessment approach for understanding acute fatigue responses following high-intensity 5 km running and may contribute to more precise training-load prescription and recovery monitoring in recreational runners.

## 1. Introduction

The performance of long-distance runners depends heavily on a complex interaction of physiological, biomechanical, neuromuscular, and psychological factors that collectively shape the athlete’s individual profile [1,2]. Within this multidimensional phenomenon, key determinants of performance include aerobic capacity, running economy, and the ability to maintain a high percentage of VO_2_max over an extended period, alongside the vital role of neuromuscular characteristics such as muscle strength and tendon stiffness [3]. High-intensity running efforts are characterized by significant physiological stress; during competitive efforts, trained runners can reach 90–100% of VO_2_max, leading to a pronounced disruption of muscle-metabolic homeostasis [4]. In this context, fatigue is not manifested solely at the muscular level through a reduced capacity to generate force but is the result of a complex interaction between peripheral and central mechanisms. Peripheral fatigue is associated with impaired muscle fiber function and the accumulation of metabolic by-products, including increased blood lactate concentration [5], while central fatigue refers to the decreased efficiency of the nervous system in activating motor units [4,6]. The investigation of these acute physiological responses gains additional importance given the massive global popularity of 5000 m races, with Parkrun serving as a prominent example. This initiative offers free, timed, weekly 5 km runs across 22 countries, attracting hundreds of thousands of participants every weekend [7]. Due to the high frequency of such regular running loads among a broad population of recreational runners, it is crucial to understand the impact of this exertion on the human body and to ensure that it does not lead to long-term adverse effects or the chronic accumulation of fatigue.

In experimental settings, the capacity to generate force production is most commonly assessed by measuring maximal voluntary isometric contraction (MVIC), which is considered a reliable indicator of changes in neuromuscular function and fatigue [8,9]. Previous research indicates that running loads of various durations and intensities can lead to a significant reduction in lower-limb isometric strength. For instance, following half-marathon running, a decrease in knee extensor MVIC was observed alongside simultaneous indicators of central and peripheral fatigue, while a pronounced decline in MVIC, particularly in men, was recorded after a high-intensity 5 km running test [4,10]. Such findings suggest that the reduction in capacity to generate force after intense running arises from the interplay of peripheral changes in the contractile apparatus and alterations in neural muscle activation. These changes can have functional consequences, including alterations in stride mechanics, which may negatively impact running economy and increase the risk of injury [11]. In addition to neuromuscular indicators, muscle architecture represents an important determinant of the capacity to generate force and the mechanical properties of skeletal muscles [12]. Since running-induced fatigue can alter spatiotemporal and kinetic running parameters, including ground contact time, stride length, and lower-limb stiffness, monitoring neuromuscular and morphological indicators can provide additional insight into the functional consequences of acute running loads [13,14].

In this context, B-mode ultrasound represents a reliable and non-invasive method for assessing the morphological and architectural characteristics of skeletal muscles, including the rectus femoris and vastus lateralis. Its application allows for the quantification of indicators such as muscle thickness, cross-sectional area, fascicle length, and pennation angle, which are frequently used to evaluate acute and chronic changes in muscle tissue [15,16]. Previous research indicates that even short-term, high-intensity loads can lead to transient changes in muscle thickness and pennation angle, further confirming the sensitivity of muscle architecture to acute mechanical stress. Such changes are most commonly associated with acute muscle swelling, alterations in fluid distribution, and the temporary morphological response of the tissue to contractile loading [17]. Therefore, ultrasonic assessment of muscle architecture can provide additional information regarding the local morphological response of the muscle, complementing data obtained through measurements of the capacity to generate force and other neuromuscular indicators. A comprehensive analysis of the organism’s response to running loads, in addition to neuromuscular and morphological parameters, includes the assessment of metabolic and perceptual load [18,19]. Blood lactate concentration is often used as an indicator of metabolic stress and the contribution of anaerobic metabolism during high-intensity work, while the rating of perceived exertion (RPE) provides insight into the perception of effort and exercise tolerance. The correlation of RPE with physiological indicators, including blood lactate and heart rate, confirms its value as a practical measure of an athlete’s internal load [20,21]. In this sense, the integration of objective and subjective indicators allows for a more holistic understanding of the physiological, metabolic, and perceptual responses of the organism to high-intensity running efforts [22].

The perception of effort plays a crucial role in regulating pacing strategies, whereby athletes continuously adjust their work intensity during performance in accordance with physiological load, previous experience, and the expected duration of the activity. Research shows that RPE, alongside physiological and muscular factors, contributes to the shaping of pacing strategies during self-paced running efforts [23,24,25]. In this context, a high-intensity 5 km run serves as an appropriate model of acute exertion for studying the neuromuscular, morphological, metabolic, and perceptual responses of the organism. Such an effort requires maintaining a high intensity within time-constrained conditions, providing an opportunity for the simultaneous analysis of changes in isometric strength, muscle morphology, blood lactate concentration, and perceived exertion. Although previous research has predominantly analyzed individual aspects of fatigue and muscle function following running bouts, few studies have monitored these indicators in an integrated manner immediately after the same protocol. Given that fatigue is a multidimensional phenomenon, the isolated monitoring of a single parameter fails to provide a comprehensive picture of the actual physiological strain on the body. An integrated analysis is essential as it enables a deeper understanding of the interactions between different mechanisms, determining the extent to which the reduction in force-generating capacity is directly driven by metabolic stress, acute morphological changes in muscle architecture, or an altered perception of effort. Such an approach provides novel theoretical insights into the specific causes of acute performance decline, while in a practical sense, it allows coaches and runners to prescribe training loads more precisely, individualize recovery strategies, and better understand fatigue mechanisms. Therefore, the aim of this study is to examine the acute neuromuscular, metabolic, and perceptual responses to a high-intensity 5 km running protocol.

## 2. Materials and Methods

### 2.1. Participants

The study involved 21 recreational male runners with multi-year experience in long-distance road running events, with an average age of 35.43 ± 11.42 years. The participants have been regularly executing structured training programs for over five years, which include aerobic long runs, tempo runs, and high-intensity interval training. All participants volunteered to take part in the study, and before the commencement of measurements, they were informed of the study’s aim and protocol and provided written informed consent. Inclusion criteria comprised a minimum age of 18 years, at least five years of continuous running experience in long-distance events, and the absence of any musculoskeletal injuries in the past six months. This ensured that the participants possessed an adequate level of training status and could safely tolerate the prescribed workload within the study. The research was conducted in accordance with the principles of the Declaration of Helsinki, and the research protocol was approved by the Ethics Committee of the Faculty of Kinesiology Osijek (Class: 029-01/25-01/05; Registry number: 2158-110-01-25-4).

### 2.2. Experimental Design

The study was conducted in November 2025 using a within-subjects pre–post experimental design to assess acute neuromuscular, metabolic, and perceptual changes following a 5 km running workload. Running trials were performed outdoors on the same athletic track in Osijek during the afternoon and evening hours and under approximately comparable seasonal outdoor conditions. All pre- and post-run measurements were conducted indoors under standardized conditions to minimize measurement-related variability. Initially, anthropometric data were collected, and body composition was assessed, followed by baseline measurements of resting blood lactate concentration. Participants then performed a standardized 10 min dynamic warm-up according to a predefined protocol. After the warm-up, baseline measurements were conducted, including resting blood lactate concentration, ultrasound assessment of right m. rectus femoris thickness, and bilateral maximal voluntary isometric contraction (MVIC) of the quadriceps.

The experimental load consisted of a 5 km run conducted on an athletic track in Osijek. Participants ran in small groups of no more than three runners, formed based on previously achieved comparable times over the same distance to ensure similar running dynamics within the groups. The pace was individually regulated but controlled, with participants instructed to maintain a high-intensity effort representative of simulated 5 km race conditions, based on their current fitness level and previous experience over this distance. Running intensity was continuously monitored using chest strap heart rate monitors synchronized with the participants’ smartwatches, and average and peak heart rate values were recorded upon completion of the run.

During the run, exercise intensity was continuously monitored using chest strap heart rate monitors synchronized with the participants’ smartwatches. Upon completion of the run, average and peak heart rate values were recorded, and the rating of perceived exertion (RPE) was assessed using the Borg CR10 scale. Immediately upon completion of the run, lactate concentration was measured, followed one minute later by an ultrasound measurement of the right m. rectus femoris thickness, while the MVIC assessment for both legs was conducted five minutes after the running protocol.

### 2.3. Measurements

Anthropometric measurements included the measurement of body height and the assessment of body composition. Body height was measured using a portable anthropometer (Harpenden Anthropometer 601, Holtain Ltd., Crymych, UK), while body composition was assessed via bioelectrical impedance analysis (BIA) using a segmental body composition analyzer (Tanita BC-601, Tanita Corporation, Tokyo, Japan). The analyzed variables included body mass (kg), body mass index (kg·m^−2^), body fat percentage (%), and muscle mass (kg). Measurements were conducted under standardized conditions with participants barefoot and wearing light clothing.

Lactate concentration was determined from capillary blood taken from the earlobe using a portable analyzer (Lactate Scout 4, SensLab GmbH, Leipzig, Germany) at rest and immediately after exercise, according to the manufacturer’s instructions, under standardized conditions and by the same experienced operator. The samples were taken after disinfection and rejection of the first drop of blood, without squeezing the injection site. Determination of lactate concentration is based on the enzymatic amperometric method and gives a result within ~10 s (range 0.5–25 mmol/L).

M. rectus femoris thickness was measured using B-mode diagnostic ultrasonography with a linear probe frequency of 5–12 MHz (set at 10 MHz). Measurement parameters were standardized (depth 3.7–4.6 cm, dynamic range 70 dB, gain 50). Measurements were performed on the right thigh at 40% of the distance between the greater trochanter and the lateral femoral epicondyle. Before measurement, participants remained in a supine position for 10 min to allow for tissue stabilization. Three images were recorded per participant, and the final value represented the mean thickness measured between the superficial and deep aponeuroses.

Maximal voluntary isometric contraction (MVIC) of the m. quadriceps femoris was assessed using an S2P isometric dynamometer (S2P, Science to Practice Ltd., Ljubljana, Slovenia). Measurements were performed in a seated position (85° angle between the trunk and thigh), with the trunk and pelvis stabilized using straps to minimize compensatory movements. The protocol consisted of three maximal isometric contractions lasting three seconds each, with a 30 s rest interval between trials. Measurements were performed bilaterally, and the highest recorded force (N) was used as the final value for each leg.

Participants were verbally asked to identify the leg they perceived as dominant or stronger during usual activities. For the purpose of side-to-side comparison, the leg with the higher baseline MVIC value was additionally classified as the stronger leg, as lower-limb dominance may vary depending on the task.

Heart rate (HR) was continuously monitored during the run using a chest strap heart rate monitor (Polar H10, Polar Electro Oy, Kempele, Finland) compatible with the participants’ smartwatches. Average and peak heart rate values were analyzed.

The rating of perceived exertion (RPE) was assessed using the validated Borg CR10 scale (range 0–10) immediately following the completion of the run. Performance was recorded as the finish time for the 5 km distance (s).

### 2.4. Statistical Analysis

Statistical data analysis was performed using jamovi software, version 2.6.44.0 (The Jamovi project, Sydney, Australia). Descriptive statistics are presented as the arithmetic mean (M), standard deviation (SD), minimum (Min), and maximum (Max) values. Data normality was assessed using the Shapiro–Wilk test. Since certain variables did not meet the assumption of normal distribution, non-parametric statistical methods were employed for further analysis.

Differences between pre- and post-exercise measurements were analyzed using the Wilcoxon signed-rank test. Effect size was expressed using the r coefficient and interpreted according to Cohen’s guidelines as small (r = 0.10), medium (r = 0.30), and large (r = 0.50). To assess the relationship between variables, Spearman’s correlation coefficient (ρ) was utilized. Only selected associations relevant to the study objectives were examined using Spearman’s rank correlation coefficient. As a sensitivity check for multiple comparisons, *p*-values were additionally adjusted using the Benjamini–Hochberg false discovery rate procedure, and the results are reported in the Supplementary Material.

A sensitivity power analysis was performed using G*Power software, version 3.1.9.4, based on the available sample size of 21 participants. The analysis indicated that the available sample size allowed detection of effects of approximately dz = 0.66 or larger for the Wilcoxon signed-rank test and correlations of approximately ρ = 0.57 or larger. Within-session reliability was assessed for repeated MVIC trials and ultrasound-derived m. rectus femoris thickness measurements. Intraclass correlation coefficients (ICC) were calculated to evaluate relative reliability, while the coefficient of variation (CV) was used to estimate absolute measurement variability. Reliability analyses were performed separately for pre- and post-run measurements. The level of statistical significance was set at *p* < 0.05.

## 3. Results

Table 1 presents participant characteristics and selected responses to the 5 km run. Participants were recreational male runners with a mean age of 35.4 ± 11.4 years and a mean body mass index of 23.3 ± 1.6 kg·m^−2^. The mean 5 km finish time was 1205 ± 238 s, corresponding to 20:05 ± 3:58 min. Mean heart rate during the run was approximately 165 bpm, while peak heart rate reached approximately 180 bpm. Together with a mean RPE value of 8.3 ± 1.2, these findings indicate that the 5 km run elicited a high cardiovascular and perceptual load.

The results of the Wilcoxon signed-rank test, which analyzed differences in blood lactate concentration (BLa), rectus femoris (RF) thickness, and MVIC before and after the 5 km run, are presented in Table 2. The maximal voluntary contraction of the right quadriceps was significantly lower post-exercise (*p* = 0.002) with a large effect size (r = 0.74), indicating a pronounced decline in force-generating capacity following the run. For the left leg, no statistically significant change in MVIC was observed (*p* = 0.103), although the effect size was moderate (r = 0.41). Blood lactate concentration significantly increased after the exercise bout (*p* < 0.001) with a very large effect size (r = 1.00), confirming substantial metabolic stress induced by the 5 km run. Overall, the findings indicate a pronounced metabolic response and a significant reduction in right-leg force-generating capacity following the 5 km run, whereas changes in left-leg MVIC and m. rectus femoris thickness did not reach statistical significance.

Within-session reliability was high to excellent for repeated measurements. MVIC measurements showed ICC values ranging from 0.851 to 0.970, with corresponding CV values ranging from 6.6% to 11.5%. Ultrasound-derived RF thickness demonstrated ICC values of 0.989 for pre-run measurements and 0.990 for post-run measurements, with corresponding CV values of 1.6% and 1.6%, respectively.

Exploratory Spearman correlation analyses were conducted for selected associations relevant to the study objectives, with detailed results provided in Supplementary Table S1. After applying the Benjamini–Hochberg false discovery rate correction for multiple comparisons, none of the selected associations remained statistically significant. Accordingly, these correlation findings should be interpreted as exploratory and hypothesis-generating.

## 4. Discussion

The aim of this study was to examine the acute neuromuscular, metabolic, and perceptual responses to high-intensity 5 km running. The objective was to obtain a comprehensive insight into the immediate post-run changes by analyzing quadriceps MVIC, blood lactate concentration, rating of perceived exertion (RPE), and m. rectus femoris thickness as an indicator of acute alterations in muscle architecture. The findings suggest that the 5 km high-intensity running stimulus resulted in a pronounced metabolic and partial neuromuscular response, whereas no significant acute changes in m. rectus femoris thickness were identified. A statistically significant increase in blood lactate concentration, accompanied by a very large effect size, confirms the substantial metabolic demand of the prescribed high-intensity 5 km running effort. Simultaneously, a statistically significant decrease in right leg quadriceps MVIC was observed with a large effect size, while the change in the left leg did not reach statistical significance. This pattern suggests that the acute reduction in force-generating capacity was more evident in the right leg. However, the side-specific nature of this response should be interpreted with caution. Limb dominance was assessed using self-reported information on the leg perceived as stronger during usual activities and was additionally considered during bilateral MVIC testing. Nevertheless, running-specific biomechanical dominance or asymmetry was not directly measured. Therefore, the observed right–left difference should be interpreted as a side-specific response rather than direct evidence of biomechanical asymmetry. Overall, the results indicate that high-intensity 5 km running induces acute responses of varying magnitudes depending on the specific indicator observed. This underscores the importance of an integrated approach in assessing acute fatigue, as individual indicators do not necessarily exhibit the same sensitivity to the same running stimulus.

### 4.1. Neuromuscular Response Following the Running Load

Furthermore, following the 5 km run, a statistically significant decrease in right-leg quadriceps MVIC of approximately 15% was observed in recreational runners, while the 9% decline in the left leg did not reach statistical significance. Although MVIC also decreased in the left leg, this change should be interpreted cautiously because it did not reach statistical significance. These findings suggest that the acute reduction in force-generating capacity was more evident in the right leg. From a biomechanical perspective, the quadriceps contribute to knee control, shock absorption, stabilization, and force transfer during the stance phase of running. Therefore, a greater reduction in right-leg MVIC may partly reflect side-specific loading during the running task. However, this interpretation should be considered cautiously, as running-specific biomechanical variables were not directly assessed [26,27]. The observed reduction in MVIC may therefore reflect an acute exercise-induced impairment in force-generating capacity, potentially involving peripheral fatigue mechanisms and, to a lesser extent, a possible neural contribution [4]. In biomechanical terms, during the ground contact phase, the quadriceps eccentrically control knee flexion and absorb mechanical energy, while during the transition to the push-off phase, it participates in stabilization and the efficient transfer of force through the lower extremity. Repetitive exposure to these demands across a high number of running cycles may result in cumulative loading of the musculoskeletal system and contribute to post-exercise reductions in force-generating capacity [26,27]. Previous research, such as the study by da Rosa et al. [28], has described functional and lateral asymmetry within stride mechanics, suggesting that the lower limbs may not contribute equally to force generation and load absorption during running. The researchers clearly state that the push-off phase involves the release of previously stored elastic energy and the generation of positive mechanical work, where the limb assumes a greater role in propulsion, simultaneously bearing a higher mechanical and metabolic load.

Nevertheless, although limb dominance was assessed by self-report and bilateral MVIC testing, running-specific biomechanical asymmetry was not directly measured and therefore remains a possible, but unconfirmed, explanation for the observed side-specific MVIC response.

Although research directly investigating the impact of MVIC on long-distance runners remains scarce, several studies support the direction of the results obtained in this study. One such study by Nummela et al. [26,29] conducted on 18 well-trained runners, tested bilateral MVIC on a specialized leg press device before and after a 5 km run and found a 15% decrease in MVIC. A similar study by Pons et al. [4] identified a significant reduction in knee extensor MVIC of 15.1% in men immediately after a 5 km run and interpreted this decrease as a consequence of both peripheral and central fatigue mechanisms. In the present study, the observed reduction in MVIC is consistent with an acute post-exercise decline in force-generating capacity, which may involve both peripheral fatigue mechanisms and changes in neuromuscular activation. Furthermore, similar studies involving MVIC measurements of other muscle groups have recorded even greater reductions. A study by Girard et al. [5] reported a decrease in plantar flexor MVIC of as much as 27% immediately following a 5 km running test, which, as in previous studies, was attributed to reduced muscle activation. The results of the present study are further supported by Taipale et al. [11], who emphasize that 5 km running loads typically reduce lower-limb isometric strength and power by about 15%, while longer durations, such as a two-hour run or combined training loads, cause a reduction in maximal force ranging from 14% to 19%. Therefore, the reduction in MVIC observed in the present study can be interpreted as an indicator of acute exercise-induced fatigue, manifested by a reduced ability to generate force after high-intensity running. Potential peripheral mechanisms may include impaired excitation–contraction coupling, altered muscle membrane excitability, and reduced calcium release from the sarcoplasmic reticulum, all of which may contribute to a reduced capacity to generate force after high-intensity running [30,31].

### 4.2. Metabolic and Perceptual Responses to the 5 km Run

In addition to the reduction in MVIC, this study recorded a statistically significant increase in blood lactate concentration following the 5 km run, indicating a markedly enhanced metabolic and energetic activation of the muscles during the effort (Table 2). The marked increase in blood lactate concentration, accompanied by high RPE values, indicates that the 5 km run elicited substantial metabolic and perceptual strain in the participants. Such a robust metabolic response, accompanied by lactate accumulation and increased intramuscular metabolic stress, may contribute to impaired contractile function and reduced force-generating capacity. These results are further supported by the research of Girard et al. [5], who state that the accumulation of metabolic by-products, such as hydrogen ions, which increase proportionally with lactate, can disrupt excitation–contraction coupling within the muscle tissue. Specifically, a drop in intracellular pH reduces the sensitivity of myofilaments to calcium and limits its release within the sarcoplasmic reticulum, thereby contributing to a reduced capacity for muscle contraction [5]. Alongside objective indicators of fatigue, we utilized the RPE scale (Rating of Perceived Exertion) to numerically assess the intensity of fatigue during physical activity. In practice, linear models such as the CR10 scale are frequently used, where workload is graded from 0 (complete rest) to 10 (maximal possible effort) [32]. This provided subjective insight into the perceived intensity of the running effort. These findings fully align with previous research indicating that, in addition to mechanical indicators of force decline, a 5 km performance induces significant metabolic and perceptual stress. Similar observations were reported by de Sousa et al. [22], who monitored physiological and perceptual responses in trained runners during a 5 km bout performed in both continuous and interval modes. In their study, parameters were monitored at the first and fifth kilometers; RPE values increased significantly as the effort progressed, reaching extremely high levels at the very end of the bout, clearly reflecting cumulative perceptual stress during the race. Conversely, blood lactate concentration at the end of the continuous race was 2.8 mmol/L, whereas in the interval race, it reached 4.5 mmol/L, indicating that interval loading caused more significant lactate accumulation compared to continuous running. Consistent with our findings, the marked increase in blood lactate concentration after the run, together with a high peak heart rate (180 ± 16 bpm), indicates substantial metabolic strain and suggests a considerable contribution of anaerobic metabolism during the 5 km effort, as also reported by Girard et al. [5]. Furthermore, the peak heart rates recorded in our research mirror the rating of perceived exertion (RPE), which was consistently high across all participants (RPE = 8.3 ± 1.2 on the CR10 scale). This suggests that participants performed the race near submaximal intensity, balancing their running speed precisely at the threshold of high-effort perception tolerance. This relationship between physiological and perceptual indicators strongly supports research highlighting RPE as a key factor in limiting tolerance to training workloads [33]. Such observations were confirmed in practice by Marcora and Bosio [19], who showed that runners with pre-induced leg muscle fatigue achieved a 4% poorer result in a 30 min race. Since cardiovascular and metabolic parameters (heart rate and lactate) remained unaffected, while the reported perception of effort was equally high despite a significantly slower pace, the authors suggested that the performance decline was not primarily explained by poorer physiological economy. This indicates that impaired muscle function may increase perceived effort and contribute to pacing adjustments during sustained high-intensity running [19].

### 4.3. Changes in M. Rectus Femoris Thickness Following a 5 km Run

Regarding the objective assessment of muscle architecture, despite the pronounced metabolic response and the significant reduction in right-leg MVIC, our study found no statistically significant change in resting m. rectus femoris thickness. Our results suggest that an acute 5 km running workload does not result in immediate measurable morphological changes in the relaxed muscle, such as cellular swelling, immediately following the cessation of activity. The observed post-run decline in force-generating capacity was not accompanied by a measurable change in rectus femoris thickness, suggesting that this specific architectural parameter did not show an immediate response to the 5 km running load. However, muscle thickness represents only one aspect of muscle architecture and may not fully characterize acute exercise-induced architectural changes. Other ultrasound-derived variables, such as fascicle length, pennation angle, cross-sectional area, and echo intensity, could potentially reveal post-exercise alterations that were not detected by rectus femoris thickness alone. Therefore, the absence of an immediate change in muscle thickness should be interpreted specifically in relation to this parameter, rather than as evidence that no acute architectural changes occurred. This interpretation is supported by Landers-Ramos et al. [34,35] who used ultrasound to examine acute changes in m. rectus femoris architecture in 11 runners and reported no immediate increase in muscle thickness after exhaustive running, with structural changes becoming evident only after approximately 24 h. This suggests that changes in rectus femoris thickness may be more evident during the recovery period rather than immediately after the race. Because the present study assessed muscle thickness only immediately after the 5 km run, potential delayed changes in rectus femoris architecture cannot be excluded. Muscle swelling, inflammatory responses, and architectural alterations may become more evident several hours after exercise or during the first 24–48 h of recovery. Therefore, future studies should include repeated ultrasound and neuromuscular assessments at multiple recovery time points, including 24 h and 48 h follow-up measurements, to better describe the time course of post-run muscle recovery. It is important to emphasize that the lack of observed changes in the m. rectus femoris is not due to insufficient sensitivity of the measurement method. More precisely, the absence of significant change should not necessarily be interpreted as a limitation of ultrasound sensitivity. On the contrary, several studies have shown that ultrasound is sensitive enough to detect morphological and functional changes in the m. rectus femoris and represents a reliable method for monitoring muscle architecture and its adaptations. For instance, Baroni et al. [35] used ultrasound to identify significant long-term morphological adaptations, such as increased muscle thickness and fascicle length, resulting from a 12-week eccentric strength training program on an isokinetic device. Furthermore, in the context of assessing muscle function, Delaney et al. [15] demonstrated the validity of ultrasound by tracking acute changes in m. rectus femoris dimensions (such as decreased width and a slight increase in thickness) during isometric contractions of varying intensities on a dynamometer. Although both studies confirmed the sensitivity of ultrasound in detecting morphological and functional changes, they were primarily conducted under strictly controlled, isolated conditions rather than immediately following a running workload. The unchanged rectus femoris thickness may therefore be related to the timing of the post-exercise assessment, the specific characteristics of the running stimulus, or the limited scope of the ultrasound assessment, which included only one architectural parameter.

#### Practical Implications and Limitations

From a practical perspective, the present findings indicate that high-intensity 5 km running may impair quadriceps force-generating capacity without producing immediately detectable changes in rectus femoris thickness. Therefore, post-run fatigue assessment should combine functional, metabolic, and perceptual indicators, such as MVIC, blood lactate concentration, and RPE, rather than relying solely on structural ultrasound measures. This integrated approach may be useful for coaches, practitioners, and researchers when monitoring acute fatigue and planning recovery after high-intensity running.

This study has several important limitations that should be considered when interpreting the results. First, the participants were not monitored over an extended recovery period, such as 24–48 h, which limits the ability to detect delayed morphological and inflammatory changes and precludes a more detailed insight into the time course of neuromuscular fatigue and recovery processes. In addition, only rectus femoris thickness was analyzed, whereas other architectural parameters, including fascicle length, pennation angle, cross-sectional area, and echogenicity, were not assessed. Second, the assessment of muscle thickness was limited to the rectus femoris and to the immediate post-race period. Therefore, potential changes in other running-related muscle groups, as well as the subsequent time course of muscular responses, remain unknown. Although the sensitivity power analysis indicated that the available sample size was sufficient to detect moderate-to-large pre–post effects and relatively large correlations, the relatively small sample size should still be considered when interpreting the findings. This is particularly relevant for non-significant findings, including changes in left-leg MVIC, rectus femoris thickness, and exploratory correlation analyses, where the possibility of Type II error cannot be excluded. The inclusion of recreational male runners only further limits the generalizability of the findings to female runners and broader athletic populations. In addition, the study did not include direct measures of central nervous system involvement, such as electromyography, twitch interpolation, or central activation ratio; therefore, the contribution of central fatigue to the observed MVIC reduction cannot be directly determined. Although limb dominance was assessed through self-report and bilateral MVIC testing, running-specific biomechanical asymmetry was not directly measured and therefore remains a possible, but unconfirmed, explanation for the observed side-specific MVIC response. Other explanations, including possible measurement variability, inter-individual variability in fatigue responses, and limited statistical power, should also be considered when interpreting the observed side-specific MVIC response. Finally, it should be noted that some of the variables employed, such as the rating of perceived exertion (RPE), depend on individual perception and can be influenced by motivational and psychological factors despite the standardized measurement procedure. Also, it is important to note that formalized protocols for determining maximal lactate steady state (MLSS) were not applied in this study.

## 5. Conclusions

High-intensity 5 km running induced pronounced metabolic and perceptual stress, as well as an acute reduction in quadriceps force-generating capacity, reflected by a significant decrease in right-leg MVIC. Although fatigue following this type of effort is well documented, the originality of this study lies in the simultaneous assessment of metabolic, perceptual, neuromuscular, and morphological indicators immediately after the run. This integrated approach provides a more comprehensive insight into the acute post-run response across different physiological and functional domains.

The lack of immediate change in rectus femoris thickness suggests that the observed reduction in MVIC was not accompanied by an acute detectable change in this architectural parameter. In addition, the significant decrease in MVIC observed only in the right leg indicates a side-specific response. However, biomechanical asymmetry remains a possible, but unconfirmed, explanation because running-specific biomechanical variables were not directly assessed.

Although the present study does not allow the underlying fatigue mechanisms to be determined directly, the findings contribute to existing knowledge by showing that different indicators do not exhibit the same sensitivity to a high-intensity 5 km running stimulus. From a practical perspective, post-run fatigue assessment should combine functional, metabolic, and perceptual indicators, including MVIC, blood lactate concentration, and RPE. In contrast, ultrasound-assessed morphological changes may be more informative for monitoring delayed recovery responses or longer-term adaptations than for detecting immediate post-run fatigue.

## Figures and Tables

**Table 1:** Participant characteristics and selected responses to the 5 km run.

Variable	M	SD	Min	Max
Age (years)	35.4	11.4	19	54
Body mass index (kg·m^−2^)	23.3	1.6	19.7	25.7
Body fat (%)	18.5	5.3	8.7	27.9
Muscle mass (%)	39.2	3.5	32.4	44.5
Mean heart rate (bpm)	165	16	131	198
Peak heart rate (bpm)	180	16	142	210
Finish time (s)	1205	238	905	1965
RPE	8.3	1.2	6	10

Note. RPE = rating of perceived exertion.

**Table 2:** Changes in m. rectus femoris thickness, MVIC, and blood lactate concentration from pre- to post-5 km run.

Variable	Pre (M ± SD)	Post (M ± SD)	W	*p*	r
BLa (mmol·L^−1^)	0.8 ± 0.4	6.9 ± 1.4	0.0	<0.001	1.00
RF thickness (mm)	17.7 ± 2.9	17.8 ± 2.9	98	0.562	0.15
MVIC R (N)	305 ± 99	259 ± 88	201	0.002	0.74
MVIC L (N)	265 ± 97	241 ± 87	163	0.103	0.41

Note. BLa = blood lactate; RF = rectus femoris; MVIC = maximal voluntary isometric contraction; R = right; L = left.

## Data Availability

The original contributions presented in this study are included in the article. Further inquiries can be directed to the corresponding author.

## Supplementary Materials

The following supporting information can be downloaded at https://www.mdpi.com/article/10.3390/sports14070262/s1, Table S1: Spearman correlations with FDR-adjusted *p*-values.
