# Supplementary material for: Indicators of Neuromuscular, Metabolic and Perceptual Fatigue Following a 5 km Run
Source: Sports (Basel). 2026 Jun 25;14(7):262. doi: 10.3390/sports14070262 (PMC13418486; doi:10.3390/sports14070262)
Supplement: Supplementary file 1 [file sports-14-00262-s001.zip › sports-4359509-supplementary.pdf]

Supplementary Table S1. Spearman correlations with FDR-adjusted p-values

Variable pair	Spearman's ϱ	Unadjusted p	FDR-adjusted p
Age – Peak HR	−0.760	<0.001	0.022
Mean HR – Peak HR	0.850	<0.001	0.022
Age – BLa Δ	−0.643	0.002	0.030
Age – Finish time	0.562	0.008	0.081
Age – Mean HR	−0.557	0.009	0.081
BMI – RF thickness Δ	−0.505	0.020	0.150
BLa Δ – Peak HR	0.488	0.025	0.161
BMI – Finish time	0.444	0.044	0.240
MVIC R Δ – Mean HR	−0.437	0.048	0.240

Note. The table includes correlations significant before multiple-comparison correction. FDR-adjusted p-values were calculated using the Benjamini–Hochberg procedure across the full correlation matrix. BMI = body mass index; BLa = blood lactate; RF = rectus femoris; MVIC = maximal voluntary isometric contraction; HR = heart rate; R = right; Δ = post–pre difference.
